# Roles of plasma proteins in mediating the causal effect of the lipid species on gastric cancer: Insights from proteomic and two-step Mendelian randomization

**DOI:** 10.1097/MD.0000000000042485

**Published:** 2025-05-16

**Authors:** Zhenhua Dong, Zhiqing Chen, Kai Yu, Dingliang Zhao, Jianling Jia, Xulei Gao, Daguang Wang

**Affiliations:** a Gastric and Colorectal Surgery Department, The First Hospital of Jilin University, Changchun, Jilin, China; b Department of Neurology and Neuroscience Center, The First Hospital of Jilin University, Changchun, Jilin, China; c Urology Department, The First Hospital of Jilin University, Changchun, Jilin, China; d Second Urology Department, The First Hospital of Jilin University, Changchun, Jilin, China; e Department of Breast Surgery, General Surgery Center, The First Hospital of Jilin University, Changchun, Jilin, China; f Second Department of Hepatobiliary and Pancreatic Surgery, The First Hospital of Jilin University, Changchun, Jilin, China.

**Keywords:** drug target, gastric cancer, lipid species, Mendelian randomization, plasma proteins

## Abstract

The change of plasma lipid species has close contacts with gastric cancer (GC). However, the specific mechanism still needs to be explored further. We aim to utilize plasma proteins to decipher the association between lipid species and GC, and seek possible drug targets for GC. We performed a two-step Mendelian randomization (MR) analysis to investigate causal relationships among 179 lipid species, 4907 plasma proteins, and GC. Using summary-data-based MR and colocalization, we first examined protein–GC associations in discovery (N = 35,559) and validation (N = 54,219) cohorts. Subsequent MR analyses assessed lipid–GC and lipid–protein relationships, followed by mediation analysis using error propagation methods. Finally, macromolecular docking of prioritized proteins identified potential therapeutic ligands. Our MR analysis revealed causal relationships between 12 lipid species and GC, as well as 3 plasma proteins and GC. Importantly, mediation analysis demonstrated that CCDC80 protein mediates 2.90% (95% CI: 0.30–5.5%) of the protective effect of diacylglycerol (16:1_18:1) against GC. Based on these findings, we identified valproic acid as a promising therapeutic candidate targeting CCDC80 for GC treatment. Our study demonstrates that reduced CCDC80 expression mediates the tumor-promoting effects of diacylglycerol (16:1_18:1) in GC pathogenesis. Molecular docking confirms valproic acid binds stably to CCDC80, suggesting its therapeutic potential. These findings advance GC etiology understanding and provide a new drug development direction.

## 1. Introduction

Gastric cancer (GC) remains a critical global health challenge, with approximately 1 million new cases diagnosed annually worldwide, ranking as the third leading cause of cancer-related mortality. This disease imposes substantial economic burdens on healthcare systems.^[1,2]^ Emerging evidence highlights the interplay between blood lipid profiles and GC risk. A prospective cohort study of 33,733 adults demonstrated that hypocholesterolemia correlates with increased cancer incidence, including GC,^[3]^ a finding corroborated by meta-analytic evidence on blood lipid–GC associations.^[4]^ Notably, a lipidomics investigation identified 11 plasma lipids inversely associated with GC development, which were further utilized to stratify risks of gastric lesion progression, enabling early identification of high-risk populations.^[5]^ Proteomic alterations also contribute to GC pathology. Elevated plasma Hsp90α levels in GC patients versus healthy controls suggest its potential as a diagnostic biomarker.^[6]^ Complementary LC-MS analyses revealed upregulated sex hormone-binding globulin expression in GC tissues.^[7]^ While plasma proteins and lipid species exhibit complex interrelationships across multiple pathologies, their causal associations remain poorly characterized. We propose a mediating role of plasma proteins in linking dysregulated lipid metabolism to GC pathogenesis. Elucidating these pathways could not only advance mechanistic understanding of gastric carcinogenesis but also inform the development of targeted therapeutic strategies.

The current standard therapeutic regimen for GC, definitive surgical resection combined with chemoradiotherapy^[8]^, is frequently associated with severe adverse effects, including nausea, leukopenia, and immunosuppression. While targeted therapies demonstrate superior tolerability profiles and hold significant clinical potential by mitigating these complications,^[9]^ their limited therapeutic diversity constrains broad clinical application. This critical gap underscores the urgent need to expand the repertoire of molecular targets. Our study addresses this challenge through a systematic investigation of blood protein–lipid interactions in GC pathogenesis, aiming to identify novel druggable targets that may inform the development of precision therapies with improved safety–efficacy balances.

Mendelian randomization (MR), an innovative epidemiological approach, enables causal inference between exposures and outcomes by leveraging genetic variants as instrumental variables (IVs) derived from genome-wide association studies (GWAS).^[10]^ The methodological rigor of MR stems from the random allocation of genetic variants during gamete formation, which minimizes confounding biases as environmental factors cannot alter germline genetic variation.^[11]^ Capitalizing on these advantages and the expanding availability of GWAS datasets, our study employs MR to mechanistically delineate plasma protein-mediated pathways linking lipidomic perturbations to gastric carcinogenesis. Furthermore, we integrate computational docking to screen therapeutic compounds targeting prioritized blood proteins, thereby bridging etiological discovery with translational drug development: a dual strategy designed to overcome current limitations in GC targeted therapies.

## 2. Methods

### 2.1. Study design

**Figure 1.**
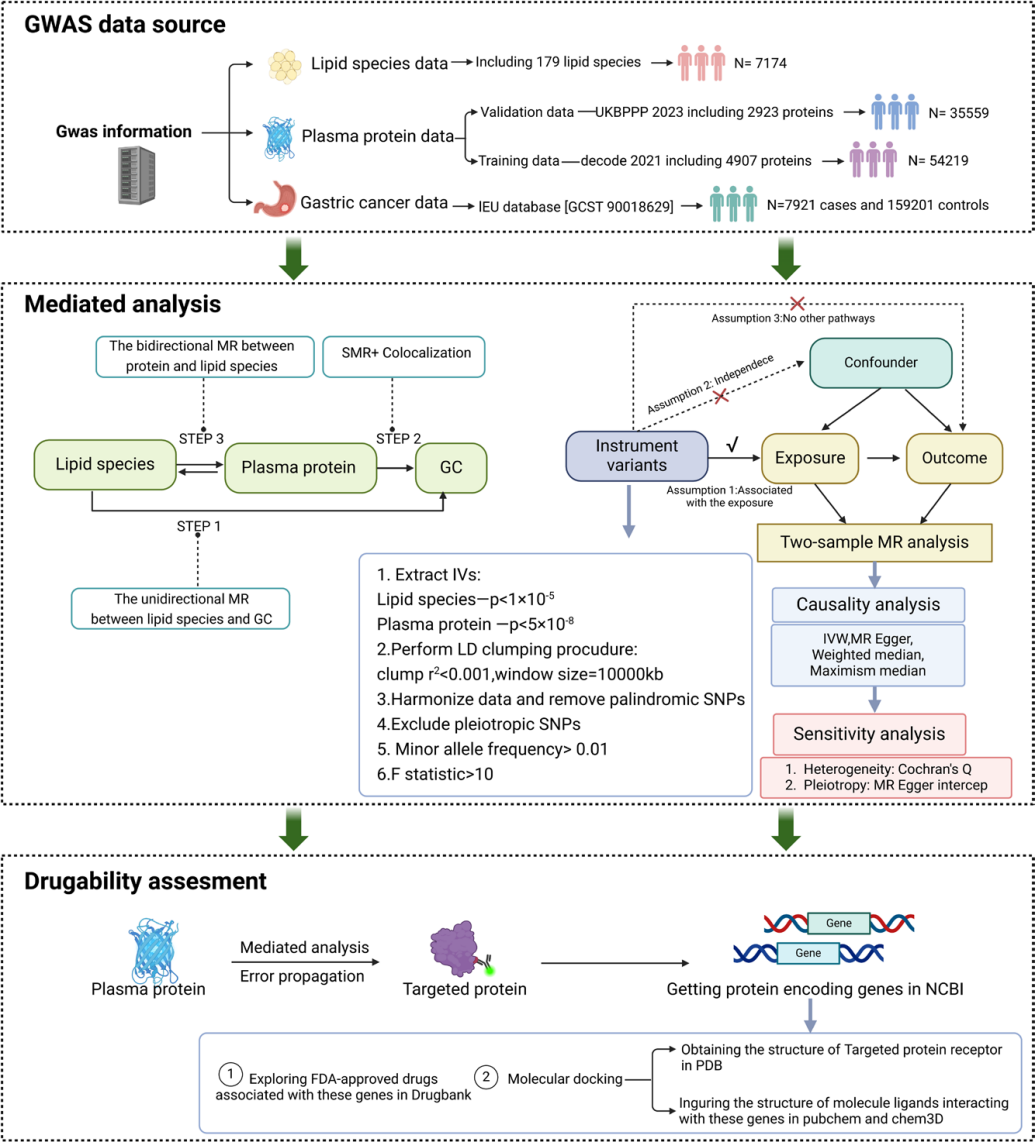
The flow chart of study. GC = gastric cancer; GWAS = genome-wide association studies; IVs = instruments variants; LD = linkage disequilibrium; MR = Mendelian randomization; NCBI = National Center for Biotechnology Information; PDB = Protein Data Bank; SMR = summary Mendelian randomization; SNP = single nucleotide polymorphism; UKBppp = UK Biobank Pharma Proteomics Project.

The flow of study is shown in Figure 1. This study rigorously adheres to the STROBE-MR guidelines, employing an integrated analytical framework across sequential phases. First, proteome-wide summary Mendelian randomization (SMR) and colocalization analyses were conducted using 2 independent datasets, the decode 2021 proteomic GWAS (N = 35,559) and the UK Biobank Pharma Proteomics Project (UKBppp 2023, N = 54,219), to identify plasma proteins causally associated with GC. Subsequently, univariable MR was performed on 179 plasma lipid species to pinpoint GC-related metabolites, followed by bidirectional MR analyses to delineate protein–lipid interaction networks. Further mediation analysis with error propagation estimation quantified pathway-specific effects. Building on prioritized mediator proteins, translational investigations were implemented: DrugBank screening identified repurposable FDA-approved drugs, while molecular docking simulations (AutoDock Vina) revealed compounds with stable binding affinities to target proteins. This pipeline bridges etiological discovery to therapeutic development, innovatively connecting causal mechanisms with actionable drug targets through multi-omics integration.

### 2.2. GWAS information

The proteomic analyses utilized 2 independent cohorts: the discovery dataset (decode2021) comprised 4907 plasma proteins quantified via SomaScan v4 assay in 35,559 Icelandic individuals (mean age = 50; sex ratio ≈ 1:1), with protein quantitative trait loci (pQTLs) identified through linear mixed modeling.^[12]^ Validation employed the UKBppp2023 cohort (N = 54,219) measuring 2923 proteins using Olink Explore 3072 platforms.^[13,14]^ For lipidomic profiling, GWAS data from 7174 Finnish participants in the GeneRISK cohort (age 45–66 years; 4642 females, 2624 males) identified 179 lipid species across 13 classes, revealing 56 lipid-associated loci including 8 novel signals through multivariable-adjusted analyses.^[15]^ The GC case–control dataset (ebi-a-GCST90018629) integrated meta-analyses of UK Biobank, FinnGen, and Biobank Japan populations (7921 cases vs 159,201 controls), predominantly of European ancestry.^[16]^ This multi-ethnic framework combines deep phenotyping with genetic epidemiology to enhance causal inference robustness.

### 2.3. Mendelian randomization

The validity of MR findings relies on satisfying 3 core assumptions: (1) strong association between IVs and exposures (F-statistic > 10), (2) independence of IVs from measured/unmeasured confounders, and (3) exclusion restriction (IVs affect outcomes solely through exposures). Genetic instruments were selected using genome-wide significance thresholds (*P* < 5 × 10⁻⁸ for serum proteins; *P* < 1 × 10⁻⁵ for lipid species), with stringent linkage disequilibrium pruning (*r*² < 0.001 within 10,000 kb windows). We calculated instrument strength using F-statistics, excluding variants with F < 10 to mitigate weak instrument bias. Primary causal estimates were derived via inverse-variance weighted (IVW) regression, supplemented by 4 sensitivity analyses: weighted median, maximum likelihood, MR-Egger, and Cochran Q statistics. These methods collectively address pleiotropy and heterogeneity, ensuring robust causal inference under violation of MR assumptions.

Many lipid traits (e.g., LDL cholesterol, triglycerides) are influenced by genetic variants with large effect sizes (e.g., APOE, LPL, FADS1/2 loci). These variants often reach stringent genome-wide significance (*P* < 5 × 10⁻⁸) in GWAS, but weaker associations (*P* < 1 × 10⁻⁵) may still represent valid instruments due to the polygenic nature of lipid metabolism. A relaxed threshold allows inclusion of more IVs while maintaining sufficient instrument strength (F-statistic > 10). Meanwhile, pQTLs typically exhibit smaller effect sizes and fewer genome-wide significant hits. A stricter threshold (*P* < 5 × 10⁻⁸) reduces the risk of weak instrument bias and horizontal pleiotropy, which are critical for MR studies of proteins where confounding pathways are more likely. Therefore, we applied distinct *P* value thresholds for IV selection: *P* < 1 × 10⁻⁵ for lipid species and the more stringent genome-wide significance threshold of *P* < 5 × 10⁻⁸ for proteins.

### 2.4. Summary-data-based MR analysis

We employed SMR to investigate causal relationships between plasma protein levels and GC risk. The SMR approach, leveraging top cis-QTLs as IV (*P* < 5 × 10⁻⁸ within ± 1000 kb gene regions), demonstrates enhanced precision over conventional MR when integrating large-scale GWAS datasets. Stringent quality control excluded single nucleotide polymorphism (SNP) with differential allele frequencies > 0.2 or strand ambiguity probabilities > .05 across reference panels, proteomic datasets, and GC GWAS.^[17]^ Pleiotropy was assessed using the heterogeneity in dependent instruments (HEIDI) test (with SMR estimates rejected at HEIDI *P* < .01 indicating confounding genetic effects. Protein signals surviving false discovery rate correction (Benjamini–Hochberg adjusted *P* < .05) underwent subsequent colocalization analysis to confirm shared causal variants between protein expression and GC pathogenesis.

### 2.5. Colocalization analysis

To determine whether genetic associations between candidate proteins and GC arise from shared causal variants, we conducted Bayesian colocalization analysis using the coloc R package. Prior probabilities were specified as follows: P_b1_ = 1 × 10 − 4*Pb*1 = 1 × 10 − 4 (SNP associated with protein only), P_b2_ = 1 × 10 − 4*Pb*2 = 1 × 10 − 4 (SNP associated with GC only), and P_b12_ = 1 × 10 − 5*Pb*12 = 1 × 10 − 5 (SNP associated with both traits). The framework evaluates 5 mutually exclusive hypotheses for SNPs in colocalized regions: (1) H_0_: no association with either trait; (2) H_1_: association with protein expression only; (3) H_2_: association with GC risk only; (4) H_3_: association with both traits through distinct causal variants; (5) H_4_: association with both traits through a shared causal variant. Posterior probabilities (PPH_x_) were calculated for each hypothesis, with PPH_4_ > 0.6 considered strong evidence of true colocalization. This stringent threshold ensures biological plausibility while controlling for pleiotropic confounding.

### 2.6. Mediated analysis

The mediation effect was quantified through a two-stage causal pathway: the product of the causal effect of lipid species on plasma protein levels and the causal effect of these proteins on GC risk. The proportion mediated was calculated as the ratio of this mediation effect to the total effect of lipids on GC. To assess statistical uncertainty, we implemented the error propagation method, which accounts for the cumulative variance introduced at each estimation step. This approach derives the standard error of the mediation effect and constructs 95% CI for the mediated proportion, based on the principle that measurement errors in preceding calculations propagate systematically through subsequent analytical stages. Rigorous application of this method ensures robust quantification of mediation pathway reliability under multivariable causal frameworks.

### 2.7. Potential therapeutic drugs prediction and molecular docking

This study employed a systematic workflow to identify potential therapeutic agents for GC: first of all, target protein-encoding genes were retrieved through the National Center for Biotechnology Information (NCBI) database. Subsequently, DrugBank was interrogated to explore FDA-approved drugs associated with these genes for potential repurposing in GC treatment. Small molecule ligands interacting with GC-related genes were identified, and their two-dimensional structures were acquired from PubChem, followed by three-dimensional conformation generation using Chem3D. Concurrently, the crystal structure of the plasma protein receptor was obtained from the Protein Data Bank (PDB). Molecular docking simulations were performed via AutoDock Vina after preprocessing receptors and ligands into PDBQT formats with defined grid parameters encompassing binding sites using AutoDock Tools. Binding energy calculations values < -5 kcal/mol indicates stable interactions, which may provide a theoretical foundation for translational investigations.

### 2.8. Ethical statement

We take full responsibility for ensuring the accuracy and integrity of this work. Any concerns regarding data validity or methodological rigor will be thoroughly investigated by our research team. As our study exclusively utilized anonymized, publicly available datasets, we did not require additional ethics approval from our institutional review board. All original studies contributing data had obtained informed consent from participants prior to data deposition, aligning with international ethical standards. We confirm strict adherence to the Declaration of Helsinki (2013 revision) throughout this investigation, prioritizing participant privacy and data security in all analytical procedures.

All analyses were conducted using R version 4.3.1 (R Foundation for Statistical Computing), with critical implementations from the following CRAN/Bioconductor packages: TwoSampleMR (v0.5.7) for MR workflows, LDlinkR (v3.1.2) for linkage disequilibrium adjustments, ggplot2 (v3.4.4) for data visualization, SMR (v1.3.1) for summary-data-based analysis, and coloc (v5.2.2) for Bayesian colocalization. This computational environment ensures reproducibility through version-controlled dependency management.

## 3. Results

### 3.1. Plasma proteins and GC

**Figure 2.**
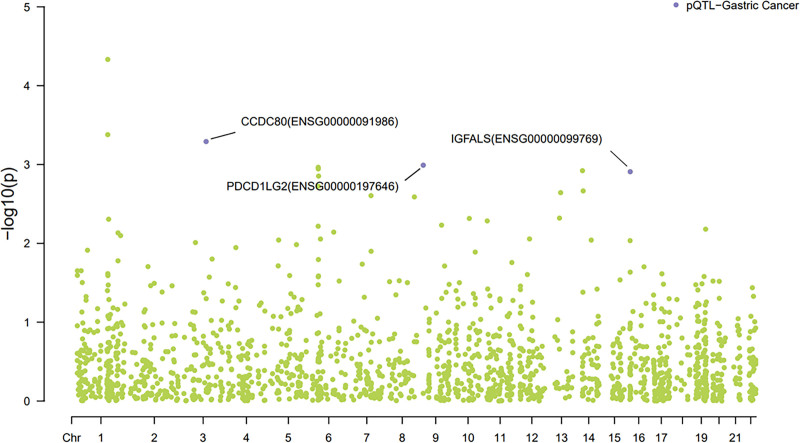
The Manhattan plot between decode proteins and GC. GC = gastric cancer.

**Figure 3.**
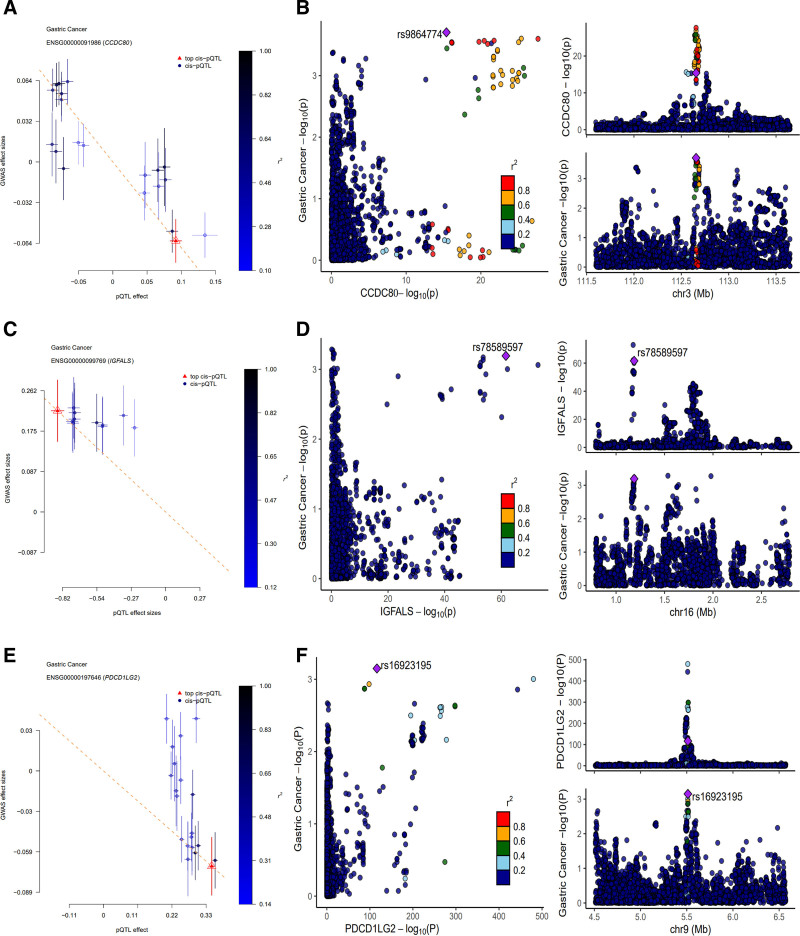
SMR scatter and colocalization locus-comparison plots between proteins and GC. (a) SMR scatter plot between CCDC80 and GC; (b) colocalization locus-comparison plots between CCDC80 and GC; (c) SMR scatter plot between IGFALS and GC; (d) colocalization locus-comparison plots between IGFALS and GC; (e) SMR scatter plot between PDCD1LG2 and GC; (f) colocalization locus-comparison plots between PDCD1LG2 and GC. GC = gastric cancer, IGFALS = insulin-like growth factor-binding protein acid-labile subunit, SMR = summary Mendelian randomization.

Proteome-wide SMR analyses identified 69 plasma proteins significantly associated with GC risk (SMR *P* < .05; HEIDI *P* > .01) in the discovery cohort (Data S1, Supplemental Digital Content, https://links.lww.com/MD/O942; Fig. 2 Manhattan plot). Among these, CCDC80 and insulin-like growth factor-binding protein acid-labile subunit (IGFALS) demonstrated strong colocalization evidence (PPH4 ≈ 0.8), while PDCD1LG2 showed moderate support (PPH4 > 0.6) (Data S2, Supplemental Digital Content, https://links.lww.com/MD/O942). Genetically predicted per SD increase in protein expression conferred protective effects against GC: CCDC80 (OR = 0.513, 95% CI = 0.139–0.889), PDCD1LG2 (OR = 0.818, 95% CI = 0.698–0.938), and IGFALS (OR = 0.774, 95% CI = 0.618–0.929). Figure 3 illustrates locus-comparison plots and SMR associations for these candidates. In validation analyses constrained by UKBppp’s limited proteome, only CCDC80 replicated at nominal significance (SMR *P* < .05, HEIDI *P* > .01; Data S3, Supplemental Digital Content, https://links.lww.com/MD/O942), though colocalization support remained absent (PPH4 < 0.6; Data S4, Supplemental Digital Content, https://links.lww.com/MD/O942), suggesting potential population-specific genetic architectures or limited statistical power.

### 3.2. 197 lipid species and GC

**Figure 4.**
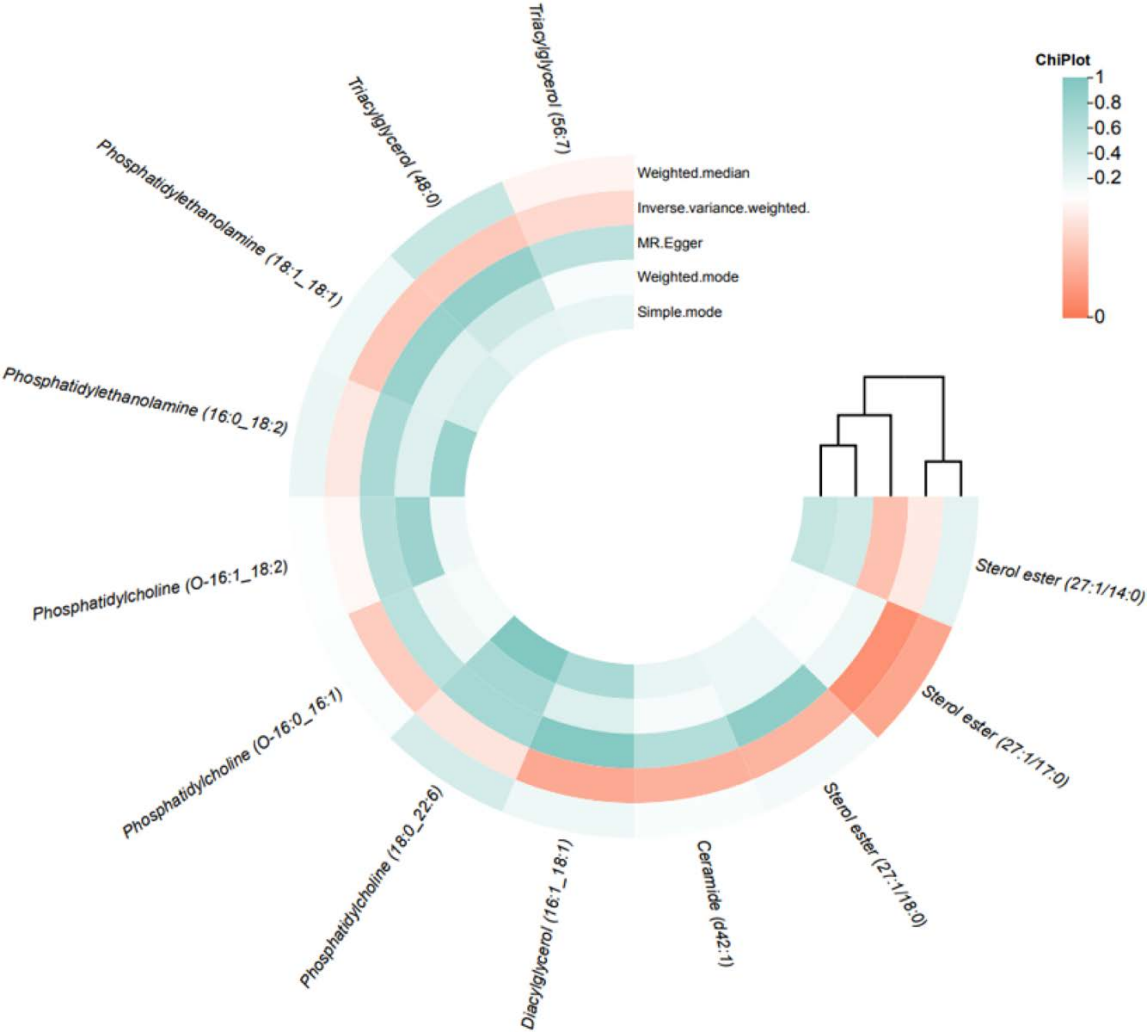
The heatmap of MR results between lipid species and GC. The legend represents the *P* value of 5 MR methods. The green indicating *P* > .05, the white indicating *P* = .05, the red indicating *P* < .05. The deeper color, the less or more the *P* value. GC = gastric cancer, MR = Mendelian randomization.

At the genome-wide significance threshold (*P* < 1 × 10⁻⁵), IVs ranging from 4 to 26 per lipid species were selected for MR analysis. IVW estimates identified 8 lipid species demonstrating significant inverse associations with GC risk: sterol ester (27:1/14:0) (OR = 0.877, 95% CI = 0.772–0.995, *P* = .042); sterol ester (27:1/18:0) (OR = 0.889, 95% CI = 0.804–0.983, *P* = .022); ceramide (d42:1) (OR = 0.913, 95% CI = 0.846–0.984, *P* = .018); phosphatidylcholine (O-16:0_16:1) (OR = 0.888, 95% CI = 0.797–0.989, *P* = .030); phosphatidylcholine (O-16:1_18:2) (OR = 0.898, 95% CI = 0.807–0.997, *P* = .047); phosphatidylethanolamine (16:0_18:2) (OR = 0.953, 95% CI = 0.910–0.998, *P* = .041); phosphatidylethanolamine (18:1_18:1) (OR = 0.927, 95% CI = 0.866–0.992, *P* = .029); triacylglycerol (56:7) (OR = 0.920, 95% CI = 0.851–0.995, *P* = .036). Conversely, 4 lipid species exhibited positive associations: sterol ester (27:1/17:0) (OR = 1.131, 95% CI = 1.031–1.242, *P* = .009); diacylglycerol (16:1_18:1) (OR = 1.102, 95% CI = 1.015–1.196, *P* = .020); phosphatidylcholine (18:0_22:6) (OR = 1.095, 95% CI = 1.004–1.194, *P* = .040); triacylglycerol (48:0) (OR = 1.078, 95% CI = 1.007–1.153, *P* = .030), as visualized in the circular heatmap (Fig. 4). Sensitivity analyses revealed no evidence of horizontal pleiotropy (MR-Egger intercept *P* > .05) or heterogeneity (Cochran Q *P* > .05) across these 12 lipid–GC associations. All IVs demonstrated robust instrument strength (F-statistics > 20), mitigating weak instrument bias (Data S5, Supplemental Digital Content, https://links.lww.com/MD/O942).

### 3.3. 12 lipid species and 3 plasma proteins

**Figure 5.**
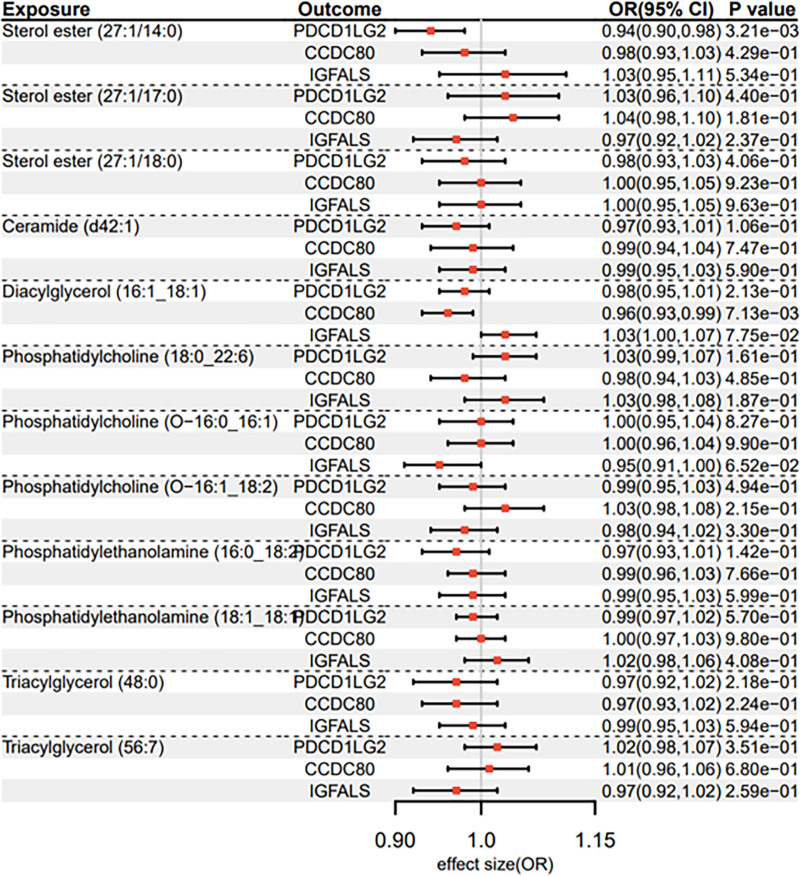
Forest plot for causal association between lipid species and proteins.

While only CCDC80 demonstrated validation cohort significance, we extended bidirectional MR analyses to 3 prioritized proteins (CCDC80, PDCD1LG2, and IGFALS) for comprehensive pathway characterization. At *P* < 1 × 10⁻⁵, robust IVs (14–24 per lipid species) were selected (Data S6, Supplemental Digital Content, https://links.lww.com/MD/O942). IVW regression revealed significant inverse associations: sterol ester (27:1/14:0) with PDCD1LG2 (OR = 0.937, 95% CI = 0.898–0.979, *P* = .003) and diacylglycerol (16:1_18:1) with CCDC80 (OR = 0.956, 95% CI = 0.925–0.988, *P* = .007) (Data S7, Supplemental Digital Content, https://links.lww.com/MD/O942; Fig. 5 Forest plot). Sensitivity analyses confirmed the absence of horizontal pleiotropy (MR-Egger intercept *P* > .05) and heterogeneity (Cochran Q *P* > .05). All instruments exceeded the minimal strength threshold (F-statistics > 10), ensuring reliable causal estimation (Data S6, Supplemental Digital Content, https://links.lww.com/MD/O942).

### 3.4. 3 plasma proteins and 12 lipid species

**Figure 6.**
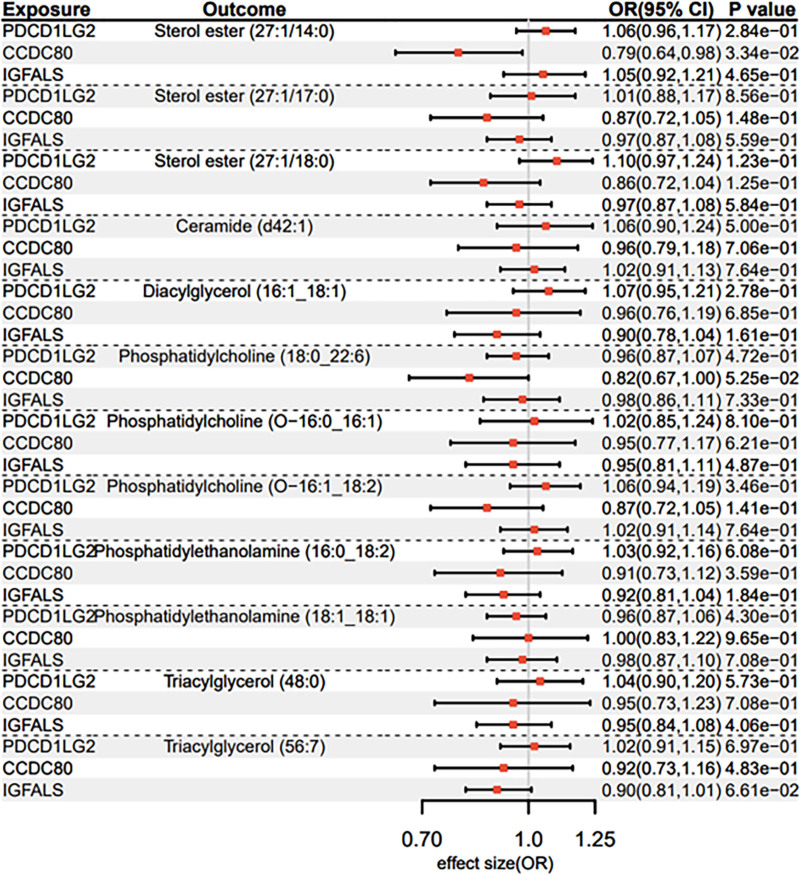
Forest plot for causal association between proteins and lipid species.

At the proteome-wide significance threshold (*P* < 5 × 10⁻⁸), IVs ranging from 6 to 10 per protein were selected for bidirectional MR analyses (Data S8, Supplemental Digital Content, https://links.lww.com/MD/O942). IVW estimates revealed a significant inverse association between genetically proxied CCDC80 levels and sterol ester (27:1/14:0) (OR = 0.790, 95% CI = 0.635–0.982, *P* = .033), as detailed in Data S9, Supplemental Digital Content, https://links.lww.com/MD/O942 and visualized in the Forest plot (Fig. 6). Sensitivity analyses confirmed no evidence of horizontal pleiotropy (MR-Egger intercept *P* > .05) or heterogeneity (Cochran Q *P* > .05). Notably, all instruments demonstrated sufficient strength for causal inference (F-statistics > 10), mitigating weak instrument bias (Data S8, Supplemental Digital Content, https://links.lww.com/MD/O942).

### 3.5. Mediated analysis

**Figure 7.**
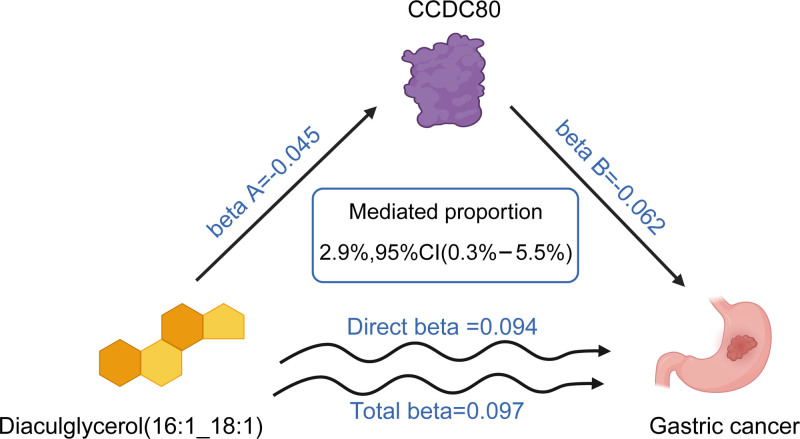
The mediated analysis of CCDC80 expression mediating the tumor-promoting effects of diacylglycerol (16:1_18:1) in GC pathogenesis. GC = gastric cancer.

Through mediation analysis, we identified 3 potential pathways: (1) diacylglycerol (16:1_18:1)–CDCC80–GC, (2) sterol ester (27:1/14:0)–PDCD1LG2–GC, and (3) CCDC80–sterol ester (27:1/14:0)–GC. For the first pathway, diacylglycerol (16:1_18:1) exhibited a total effect on GC with β = 0.097, mediated partially through CDCC80 (direct effect: lipid→protein β = -0.045; protein→GC β = -0.062), yielding a small but statistically significant mediation effect (β = 0.003, *P* = .031), accounting for 2.90% of the total effect (95% CI: 0.30–5.5%). In contrast, pathways 2 and 3 failed to demonstrate statistically significant mediation via the propagation of error method, likely due to conflicting directional effects, both total and direct effects were simultaneously negative (pathway 2: total effect β = -0.132 vs direct effects β = -0.065 and β = -0.070; pathway 3: total effect β = -0.062 vs direct effects β = -0.236 and β = -0.132), thereby violating the additive mediation assumption (Fig. 7 and Table 1).

**Table 1:** The mediation effect of lipid species on GC via plasma proteins and plasma proteins on GC via lipid species in the discovery phase.

	Total effect β	Direct effect A β	Direct effect B β	Mediation effect β	*P*	Mediated proportion (%) (95% CI)
Diacylglycerol (16:1_18:1)–CDCC80–GC	0.097	-0.045	-0.062	0.003	.031	2.9 (0.30, 5.50)
Sterol ester (27:1/14:0)–PDCD1LG2–GC	-0.132	-0.065	-0.070	0.005	.028	-3.4 (-0.40, -6.50)
CCDC80–sterol ester (27:1/14:0)–GC	-0.062	-0.236	-0.132	0.031	.259	50.0 (-36.8, 136.7)

For the first association, the term “total effect” denotes the impact of lipid species on GC. “Direct effect A” signifies the influence of lipid species on plasma proteins. “Direct effect B” represents the influence of plasma proteins on GC. The term “mediation effect” denotes the impact of lipid species on GC via plasma proteins. For the final 2 associations, the term “total effect” denotes the impact of plasma proteins on GC. “Direct effect A” signifies the influence of plasma proteins on lipid species. “Direct effect B” represents the influence of lipid species on GC. The term “mediation effect” denotes the impact of plasma proteins on GC via lipid species.

CI = confidence interval, GC = gastric cancer.

### 3.6. Molecular docking

**Figure 8.**
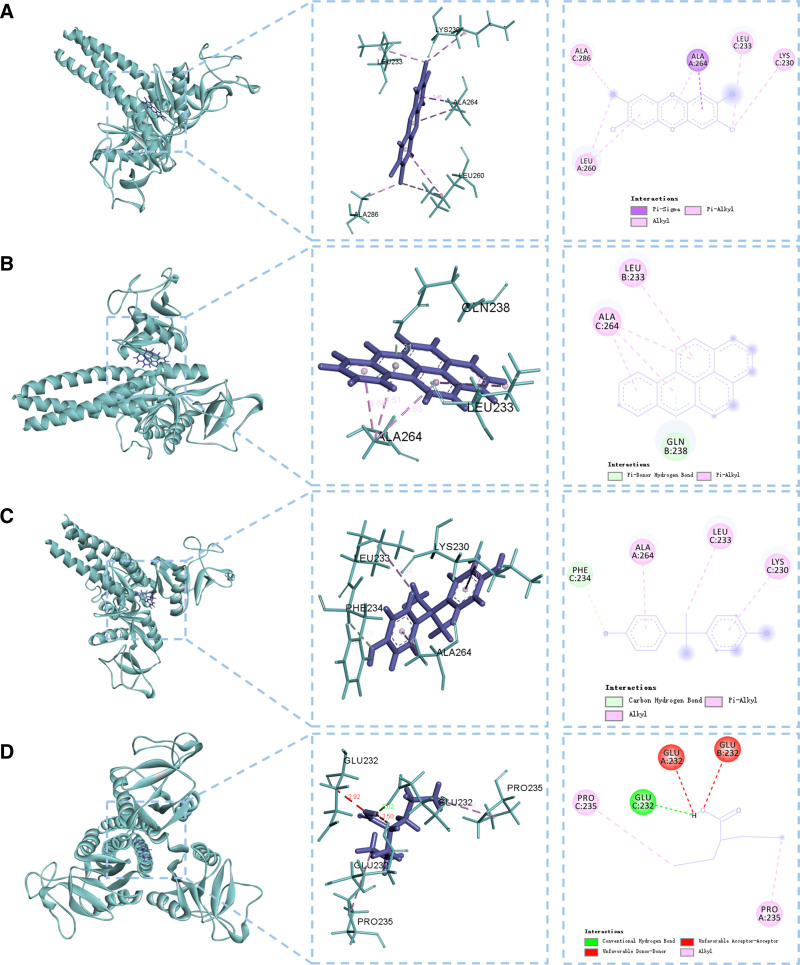
Macromolecular docking plots between components and CCDC80. (a) Macromolecular docking plots between 2,3,7,8-tetrachlorodibenzo-P-dioxin and CCDC80; (b) macromolecular docking plots between benzo[a]pyrene and CCDC80; (c) macromolecular docking plots between bisphenol A and CCDC80; (d) macromolecular docking plots between valproic acid and CCDC80.

In DrugBank, we cannot find any approved drug for GC treatment. Macromolecular docking analysis identified CCDC80 (PDB ID: 2GGU) as a potential drug target for GC. Four compounds were screened for interaction with CCDC80: 2,3,7,8-tetrachlorodibenzo-P-dioxin (PubChem ID: 15625) binds to LYS230, LEU233, LEU260, ALA264, and ALA286 with a binding energy of -5.5151 kcal/mol; bisphenol A (PubChem ID: 6623) interacts with LEU223, GLN238, and ALA264 (-5.1477 kcal/mol); benzo[a]pyrene (PubChem ID: 2336) engages LYS230, LEU233, PHE234, and ALA264 (-5.0077 kcal/mol); valproic acid (PubChem ID: 3121) binds to GLU232 and PRO235 (-4.9364 kcal/mol). All ligands exhibited binding energies near -5 kcal/mol, suggesting stable interactions. However, 2,3,7,8-tetrachlorodibenzo-P-dioxin, bisphenol A, and benzo[a]pyrene, known carcinogens or environmental hazards, were excluded due to safety concerns. Valproic acid, a regulated medication with therapeutic potential despite risks of misuse, was prioritized as the sole viable candidate for further evaluation in GC treatment (Fig. 8).

## 4. Discussion

In this study, we systematically investigated the molecular mechanisms underlying GC pathogenesis through integrated multi-omics approaches. Proteomic MR (SMR) and colocalization analyses initially identified 3 genetically predicted plasma proteins significantly associated with GC risk. Subsequent MR analyses of 179 lipid species revealed 12 plasma lipid metabolites exhibiting causal relationships with GC susceptibility. Bidirectional MR analyses further elucidated the complex interplay between dysregulated lipid metabolism and plasma protein alterations. Through two-step MR and causal mediation analysis, we demonstrated that CCDC80 protein mediates 2.90% (95% CI: 0.30–5.5%) of the association between diacylglycerol (16:1_18:1) and GC risk. Computational docking analysis identified valproic acid as a potential therapeutic agent targeting CCDC80, showing favorable binding energy (-4.94 kcal/mol) through interactions with GLU232 and PRO235 residues. This work provides novel insights into the protein–lipid axis in GC development and offers translational implications for targeted therapeutic development. Our findings not only deepen the understanding of GC pathogenesis but also establish a methodological framework for drug repurposing through genetic prioritization and computational validation.

The causal relationship between 3 prioritized proteins and GC aligns with established biological evidence. CCDC80, encoding a secreted protein containing the P-DUDES domain, regulates peroxide metabolism and tumor-suppressive signaling.^[18]^ Targeted proteomic profiling demonstrates elevated plasma CCDC80 levels in GC patients versus healthy controls (AUC = 0.87, 95% CI = 0.79–0.94), supporting its diagnostic potential.^[19]^ Mechanistically, CCDC80 mediates extracellular matrix remodeling and cell adhesion while exhibiting pan-cancer inhibitory effects: validated in colon,^[20]^ pancreatic/thyroid,^[21,22]^ and ovarian cancers. These multidisciplinary findings corroborate our MR-derived causal inference regarding CCDC80’s protective role in gastric carcinogenesis. While no prior studies have investigated IGFALS in GC pathogenesis, emerging evidence suggests its broader oncological relevance. IGFALS, a critical regulator of growth factor bioavailability and cellular metabolism,^[23]^ demonstrates tumor-suppressive properties in hepatocellular carcinoma.^[24]^ Our MR analyses reveal a novel protective association between genetically predicted IGFALS levels and GC risk, marking the first evidence of its potential antineoplastic role in gastrointestinal malignancies. This finding underscores the need for functional validation through in vitro/vivo models to delineate IGFALS-mediated mechanisms in gastric carcinogenesis and assess its therapeutic targeting potential. PDCD1LG2 (programmed death-ligand 2), a key immune checkpoint regulator, exhibits context-dependent roles in tumor biology. While it typically promotes tumor progression through immune evasion – evidenced by elevated PDCD1LG2 expression in GC tissues correlating with immunosuppressive microenvironments and chemoresistance^[25]^ – emerging preclinical studies reveal paradoxical immunostimulatory effects. Liu et al demonstrated PDCD1LG2 overexpression enhances CD8^+^ T-cell infiltration and cytotoxicity in murine GC models,^[26]^ while another study found PDCD1LG2-expressing tumor cells activate T-cell lymphokine production via specific antigen presentation modes.^[27]^ This functional duality underscores its complex immunomodulatory mechanisms. Our MR-derived protective association suggests PDCD1LG2 may exert tumor-suppressive effects in GC pathogenesis under certain genetic or microenvironmental contexts, challenging conventional oncogenic paradigms and warranting mechanistic exploration.

The complex interplay between the 12 lipid species and GC pathogenesis warrants mechanistic elucidation. Diacylglycerol, a key lipid second messenger, transduces extracellular stimuli into intracellular signaling cascades. Dysregulation of diacylglycerol signaling, whether through aberrant activity levels or cellular abundance, has been implicated in tumorigenesis, metastatic progression, and immune evasion across malignancies.^[28]^ Notably, diacylglycerol critically modulates T-cell receptor signaling, maintaining adaptive immune surveillance capabilities essential for antitumor responses.^[28]^ Supporting our MR-derived protective association for diacylglycerol (16:1_18:1), preclinical studies demonstrate therapeutic potential: Bae et al^[29]^ reported that cationic nanoparticle-mediated diacylglycerol delivery selectively induces oxidative stress-mediated apoptosis in tumor cells. These converging lines of evidence position diacylglycerol metabolism as a promising target for GC interception strategies. Phosphatidylcholine demonstrates significant associations with GC pathogenesis. Comparative lipidomic profiling using imaging mass spectrometry reveals elevated lysophosphatidylcholine acyltransferase 1 expression in GC tissues, which catalyzes enhanced phosphatidylcholine biosynthesis.^[30]^ Complementary analyses employing nanoflow ultrahigh-performance liquid chromatography-electrospray ionization-tandem mass spectrometry further identify distinct phosphatidylcholine profiles in plasma samples from GC patients versus healthy controls.^[31]^ These findings collectively suggest phosphatidylcholine metabolism may serve as both a potential biomarker and therapeutic target in GC. Ceramide, a central signaling molecule within the sphingolipid rheostat, plays dual roles in GC pathogenesis. As bioactive mediators of cellular proliferation and apoptosis, ceramide metabolic perturbations (e.g., altered ceramide synthase activity) have been mechanistically linked to gastric oncogenesis.^[32]^ Notably, ceramides exert tumor-suppressive effects through autophagic flux modulation and intrinsic apoptosis pathway activation, positioning sphingolipid metabolism as a promising therapeutic frontier.^[33]^ These pleiotropic functions underscore ceramide’s potential as both a diagnostic biomarker and molecular target for GC interception strategies.

CCDC80 likely mediates diacylglycerol (16:1_18:1)-linked GC progression through coordinated regulation of signaling pathways, epigenetic modifications, and tumor microenvironment remodeling, positioning it as a central hub in lipid-driven oncogenesis. The diacylglycerol (16:1_18:1)–CCDC80 oncogenic axis consists of 2 key steps: (1) diacylglycerol suppresses CCDC80 expression, and (2) CCDC80 deficiency promotes gastric carcinogenesis. For first step, possible mechanism may involve: (1) diacylglycerol acts as a second messenger to activate protein kinase C, which phosphorylates downstream transcriptional repressors, thereby suppressing transcription of the CCDC80 gene.^[34]^

(2) Diacylglycerol upregulates histone deacetylase activity, diminishing H3K9/H3K27 acetylation at the CCDC80 promoter region and thereby suppressing its transcriptional activity.^[35]^ (3) Diacylglycerol activates NF-κB/STAT3 signaling pathways to upregulate oncogenic miRNAs (e.g., miR-21, miR-155), which directly target CCDC80 mRNA for destabilization.^[36]^ For second step, possible mechanism may involve: (1) CCDC80 deficiency alleviates suppression of Wnt ligands (e.g., Wnt3a), enabling β-catenin nuclear translocation and activation of proliferation-driving genes (c-Myc, Cyclin D1), thereby fueling tumor cell growth.^[37]^ (2) CCDC80 suppresses epithelial–mesenchymal transition by stabilizing E-cadherin. Its loss triggers cadherin switching (reduced E-cadherin, elevated N-cadherin/vimentin), thereby enhancing cellular migratory and invasive capacities.^[38]^ (3) CCDC80 facilitates M2-to-M1 macrophage polarization through IL-4/IL-13 secretion. Its deficiency drives immunosuppressive reprogramming characterized by CD206 + M2-like tumor-associated macrophages accumulation and impaired T-cell cytolytic activity.^[39]^

This study demonstrates several key strengths. First, the rigorous analytical framework integrates multi-omics data with robust causal inference methods. Second, leveraging large-scale, cross-ancestry GWAS datasets enhances the comprehensiveness of the MR findings. Third, extensive sensitivity analyses (including weighted median, MR-Egger, and Cochran Q tests) ensure result stability. Fourth, the study systematically evaluates thousands of proteins and hundreds of lipid species, providing a panoramic view of molecular associations with GC. Fifth, the complementary application of MR, SMR, and colocalization analyses strengthens causal inference through methodological triangulation. However, certain limitations warrant consideration. First, the predominantly European ancestry of the proteomic and lipidomic GWAS data may limit the generalizability of findings to other populations. Second, potential unmeasured pleiotropy or GWAS quality constraints could affect the detection of true lipid–protein–GC associations. Third, age- and sex-stratified analyses were precluded by insufficient GWAS subgroup data. While MR provides robust evidence for causal relationships, further experimental and clinical validation is needed to confirm these observations and elucidate underlying mechanisms.

## 5. Conclusions

Our study demonstrates that reduced CCDC80 expression mediates the tumor-promoting effects of diacylglycerol (16:1_18:1) in GC pathogenesis. Molecular docking confirms valproic acid binds stably to CCDC80, suggesting its therapeutic potential. These findings advance GC etiology understanding and provide a new drug development direction.

## Acknowledgments

Thank you for all the patients in this research; thank you for all the scholars in this article. Thank you for all the teammates for supporting this research. We are also particularly grateful to our colleagues at The First Affiliated Hospital of Jilin University for their contributions.

## Author contributions

**Conceptualization:** Zhiqing Chen, Jianling Jia.

**Formal analysis:** Zhenhua Dong, Dingliang Zhao, Xulei Gao.

**Investigation:** Daguang Wang.

**Methodology:** Zhenhua Dong, Dingliang Zhao, Jianling Jia.

**Software:** Zhiqing Chen.

**Supervision:** Xulei Gao.

**Validation:** Kai Yu, Daguang Wang.

**Visualization:** Kai Yu, Daguang Wang.

## Supplementary Material
